# Effects of Compound Probiotics on Cecal Microbiome and Metabolome of Shaoxing Duck

**DOI:** 10.3389/fmicb.2021.813598

**Published:** 2022-01-11

**Authors:** Hanxue Sun, Xizhong Du, Tao Zeng, Shenggang Ruan, Guoqin Li, Zhengrong Tao, Wenwu Xu, Lizhi Lu

**Affiliations:** ^1^Animal Husbandry and Veterinary Institute, Zhejiang Academy of Agricultural Sciences, Hangzhou, China; ^2^College of Animal Science and Technology, Anhui Agricultural University, Hefei, China; ^3^Jinhua Academy of Agricultural Sciences, Jinhua, China; ^4^Shaoxing Xianheng Shao Duck Breeding Co., Ltd., Shaoxing, China

**Keywords:** compound probiotics, duck, intestinal microflora, 16S rRNA, metabolome

## Abstract

This experiment was conducted to investigate the effects of compound probiotics on intestinal microflora and metabolome of Shaoxing ducks. A total of 640 1-day-old Shaoxing ducks were randomly divided into two treatments with eight replicates and forty ducks for each replicate. The ducks were fed basal diet (Ctrl) and basal diet supplemented with 0.15% compound probiotics (MixP). The experiment lasted for 85 days. The results showed that the abundance of *Bacteroidetes* and *Bacteroides* in MixP was higher than that in Ctrl (*P* < 0.05). However, the abundance of *Firmicutes* and *Oscillospira* and *Desulfovibrio* in MixP was lower than that in Ctrl (*P* < 0.05). Concentrations of 71 metabolites differed significantly (*P* < 0.05) between the MixP and the Ctrl groups; for example, Pyridoxal (Vitamin B6), L-Arginine, and Betaine aldehyde were up-regulated (*P* < 0.05), and 7-oxocholesterol, 3-hydroxy-L-kynureni-ne, and N-acetyl-d-glucosamine were down-regulated (*P* < 0.05). KEGG was enriched in 15 metabolic pathways. The pathways of Vitamin B6 metabolism, Vascular smooth muscle contraction, Vitamin digestion and absorption, and Protein digestion and absorption were influenced by compound probiotics supplementation. Thus, supplementation of compound probiotics improved cecal heath through shifts in the cecal microbiome and metabolome.

## Introduction

The microbes in the intestinal tract of animals are composed of bacteria, archaea, and fungi. The number of microbes can be 1–10 times the total number of host cells, and the total number of genes is 100 times that of the host (Sender et al., 2016). Gut microbes colonize and coevolve with the host at birth, forming a complex ecosystem known as the gut microbiome (Bäckhed et al., 2004). Studies have shown that the diversity of flora in an animal’s digestive tract can reflect the digestive and absorption capacity of the animal, and intestinal flora plays a key role in maintaining intestinal function (Psscale et al., 2018). In addition, normal intestinal flora can promote the development and maturity of the host immune system and maintain the healthy state of the host (Fang et al., 2019). Gut microbiota are crucial for animal health and development by aiding digestion, regulating the immune system (Nicholson et al., 2012), and preventing pathogen invasion (Franchi et al., 2012). Probiotics are mainly through colonization in the host, adjust the immune function or host mucous membrane and system regulating intestinal flora balance; they promote nutrient absorption maintain intestinal health (Hill et al., 2014).

*Bacillus subtilis* is a Gram-positive probiotic bacterium, and the vast probiotic potential of *B. subtilis* has been recently demonstrated in numerous host organisms under different environmental conditions (Duanis-Assaf et al., 2020). *B. licheniformis* is widely used in industry, horticulture, and pharmacy. It is a promising producer of enzymes, platform chemicals (Petrova et al., 2020), antibiotics (Cheng et al., 2021), and EPSs with antimicrobial, antioxidant, and anticancer activities (Xu et al., 2019; Petrova et al., 2021; Vinothkanna et al., 2021). Probiotics are widely regarded as one of the most effective alternatives to antibiotics because they maintain or restore normal microbiome, inhibit pathogen adhesion to intestinal wall, prevent inflammation, and protect intestinal barrier function (Van Baarlen et al., 2013; Cisek and Binek, 2014; Gresse et al., 2017). The non-specific immunity of animals is enhanced after the use of probiotics, the growth performance of animals is improved, and there are no super drug-resistant bacteria and other phenomena, reducing the pollution to the environment (Sarangi et al., 2016). Shaoxing ducks (*Anas platyrhynchos*), as a famous egg-laying duck breed, are raised in large numbers in China (Zhao et al., 2019; Tian et al., 2020). Probiotics are of increasing interest in the poultry industry, because in many countries producers can no longer use antibiotics as growth factors (Nava et al., 2005). In this study, we focused on evaluating the effects of probiotics on intestinal microflora and metabolome of Shaoxing ducks.

## Materials and Methods

### Experimental Design

A total of 640 1-day-old Shaoxing ducks with similar body weight and good health were randomly divided into two treatments with eight replicates and forty ducks for each replicate. The control group (Ctrl) was fed a basal diet, and the compound probiotics group (MixP) was fed 0.15% probiotics on the basis of the control group. The experiment lasted for 85 days.

### Experimental Materials

Probiotic preparation (composed of *bacillus subtilis* and *bacillus licheniformis*), containing effective functional bacteria count ≥ 6.0 × 10^8^ CFU/g, provided by Guangzhou Hengyi Biotechnology Co., Ltd. The ducks were provided by Xianheng Shaoxing Duck Breeding Co., Ltd.

### Experimental Rations and Feeding Management

Diet preparation and nutrient content calculation were based on the Shaoxing duck feeding standard of China (NY/T 827-2004), and combined with the physiological and nutritional requirements of young ducks in the brooding period and early growth period, diets were formulated as shown in Table 1. The experiment was carried out in a duck factory in Shaoxing, Zhejiang Province. The duck house was cleaned regularly and the death of test ducks recorded.

**TABLE 1:** Composition and nutrient levels of the basal diet (as-dry basis) %.

Ingredients	1–28 days	28–85 days	Nutrient levels*^b^*	1–28 days	28–85 days
Corn	56.64	58.05	ME (MJ/kg)	11.37	10.97
Soybean meal	29.70	26.54	CP	19.31	16.14
Wheat bran	5.10	7.00	Lys	0.98	0.77
CaHPO_4_	1.37	1.31	Met	0.43	0.31
L-Lys	0.49	0.49	Met + Cys	0.73	0.61
DL-Met	0.20	0.16	Ca	0.94	0.81
Nacl	0.30	0.30	TP	0.65	0.40
Limestone	3.50	3.45			
Soybean oil	1.70	1.70			
Premix*^a^*	1.00	1.00			
Total	100.00	100.00			

*^a^The premixed feed was provided with 12 mg Cu, 84 mg Zn, 110 mg Mn, 10.4 mg I, 0.3 mg Se, 11 250 IU VA, and 3,000 IU VD_3_ per kg of diet. 37. 5 mg VE, 3 mg VK, 4 mg VB_1_, 7.2 mg VB_2_, 60 mg niacin, 14 mg calcium ubiquitin, 4 mg VB_6_, 1.3 mg VB_11_, 0. 02 mg VB_12_, 0.1 mg choline.*

*^b^Nutrient levels was a calculated using NRC (1994) values.*

### Sample Collection

One ducks per replicate were chosen at the end of the experiment (day 85) based on their average weight and then euthanized using carbon dioxide inhalation (birds chosen for microbiota analysis were identical to those for LSI analysis) (Cheng et al., 2021); the contents of duck cecal were collected from each duck. Then quickly frozen in liquid nitrogen, sent to the laboratory and stored at −80°C for further analysis of microbiota and metabolites.

### Intestinal Microbiome

#### Bacterial Genomic DNA Extraction and PCR Amplification

The DNA was extracted from the intestinal contents using the QIAamp rapid DNA template mini-kit (Qiagen, Hilden, Germany), and the DNA concentration and purity were monitored on a 0.8% agarose gel, which was diluted to 1 ng/μL with sterile water according to the concentration.

All PCR reactions were performed using DNA (20 ng) 1 μL, F-primer/R-primer (20 μmol/L) 0.4 μL,2 × Phusion High-Fidelity PCR Master Mix with GC Buffer [New England Biolabs (Beijing) Ltd., China] 10 μL and nucleotide-free water 8.2 μL, with thermal cycling conditions consisting of initial denaturation at 94°C for 3 min, followed by 35 cycles of 94°C for 45 s, 50°C for 60 s, and 72°C for 90 s, with a final extension step at 72°C for 10 min. Following separation using 2% agarose gel electrophoresis (in TAE buffer), PCR products in the bright main strip between 400 and 450 bp were mixed in equidensity ratios and purified with a Qiagen Gel Extraction Kit (QIAGEN, Dusseldorf, Germany).

#### Preparation of Sequencing Library and High-Throughput Sequencing

The library was constructed according to the TruSeq^®^ Nano DNA LT Library Prep Kit (Illumina, San Diego, United States), and the V3-V4 regions of 16S rDNA were amplified with specific primers (338F/806R):

338F: 5′-ACT CCT ACG GG AGG CAG CAG-3′
806R: 5′-GGA CTA CHV GGG TWT CTA AT-3′

Based on the manufacturer’s recommendations, the TruSeq^®^ DNA PCR-sample-free preparation kit (Illumina, San Diego, United States) was used to generate the sequencing library, and the barcode was added. The Illumina MiSeq platform was used for sequencing, and 250 bp paired-end readings were generated. Library construction and sequencing were performed by Shanghai Partheno Biotech Co., Ltd.

### Sequencing Data Analysis

#### Processing of Original Double-Ended Sequencing Data

Original sequencing was performed using Cut-adapt shear low-quality reads (V1.9.1).^1^ The barcode was then removed with the primer sequences, and preliminary quality control was conducted to obtain the original data (raw reads). The original data sequence (UCHIME Algorithm)^2^ (Edgar et al., 2011) was compared with the species annotation database to detect chimera sequences, which were then removed to obtain the final valid data (clean reads).

#### Classification and Classification of Operational Taxonomic Units

Operational taxonomic units (OTUs) or amplicon sequence variants (ASVs) clustering was performed based on 97% similarity using Uparse v7.0.1001.^3^ The Mothur method was used with SILVA DE^4^ (Wang et al., 2007) and the Greengenes database (Release 13.8)^5^ (DeSantis et al., 2006) of the annotated OTUs were used to represent the species sequence analysis, with a threshold of 70% used to ensure the accuracy of the results of the analysis. The OTUs with abundances less than 0.001% of the total sequencing volume of the whole sample were removed (Bokulich et al., 2013).

### LEfSe (LDA Effect Size) Analysis

Discriminant analysis is a method that combines the non-parametric Kruskal–Wallis and Wilcoxon rank sum tests with Lineardiscriminant analysis (LDA) combined with Effect size (Segata et al., 2011). LEfSe analysis is a difference analysis method, which can directly analyze the difference of all classification levels at the same time. Meanwhile, LEfSe put more emphasis on finding robust differences between groups, namely biomarkers.

### Intestinal Metabolomics

Metabolomics analysis based on LC-MS. After the samples were thawed slowly at 4°C, samples were added to pre-cooled acetonitrile/aqueous solution (2:2:1, V/V), followed by vortex mixing, low-temperature ultrasound for 30 min, standing at −20°C for 10 min, and 14,000 g centrifugation at 4°C for 20 min. The supernatant was vacuum dried, and 100 μL acetonitrile aqueous solution (acetonitrile: Water = 1:1, V/V) redissolved, vortex, and 14,000 g centrifuged at 4°C for 15 min, and the supernatant was sampled for analysis.

Metabolic extracts were analyzed four times using HILIC and RPLC separation in both positive and negative ionization modes. Data were acquired on a Q Exactive plus mass spectrometer for HILIC and a Q Exactive mass spectrometer for RPLC (Thermo Scientific, San Jose, CA, United States). Both instruments were equipped with a HESI-II probe and operated in full MS scan mode. MS/MS data were acquired on quality control samples (QC) consisting of an equimolar mixture of all samples in the study. HILIC experiments were performed using a ZIC-HILIC column 2.1 × 100 mm, 3.5 μm, 200Å (cat# 1504470001, Millipore, Burlington, MA, United States), and mobile phase solvents consisting of 10 mM ammonium acetate in 50/50 acetonitrile/water (A) and 10 mM ammonium acetate in 95/5 acetonitrile/water (B). RPLC experiments were performed using a Zorbax SBaq column 2.1 × 50 mm, 1.7 μm, 100Å (cat# 827700–914, Agilent Technologies, Santa Clara, CA, United States) and mobile phase solvents consisting of 0.06% acetic acid in water (A) and 0.06% acetic acid in methanol (B). Data quality was ensured by (i) injecting 6 and 12 pool samples to equilibrate the LC-MS system prior to run the sequence for RPLC and HILIC, respectively, (ii) injecting a pool sample every 10 injections to control for signal deviation with time, and (iii) checking mass accuracy, retention time, and peak shape of internal standards in each sample (Contrepois et al., 2020).

### Statistical Analysis

The statistical calculations assessing bacterial community, metabolites, and their correlation data were carried out by conducting tests using the SPSS software package (SPSS version 23.0; IBM Corp., Armonk, NY, United States). The Kruskal–Wallis rank sum test was used to select and demonstrate differentially abundant taxa between the groups. The first principal component with a variable importance in the projection value > 1.0 and a *P*-value < 0.05 in Student’s *t*-test were considered to be significantly different metabolites. The correlations between different cecal microbial genera (*P* < 0.05 and relative abundance > 0.05% in at least one of the samples) and various altered cecal metabolites were assessed by Spearman’s correlation test. Significance was declared at *P* < 0.05, and a tendency was declared at 0.05 ≤ *P* < 0.10.

## Results

### Bacterial Analyses of Cecal Contents

#### Data Summary

**FIGURE 1:**
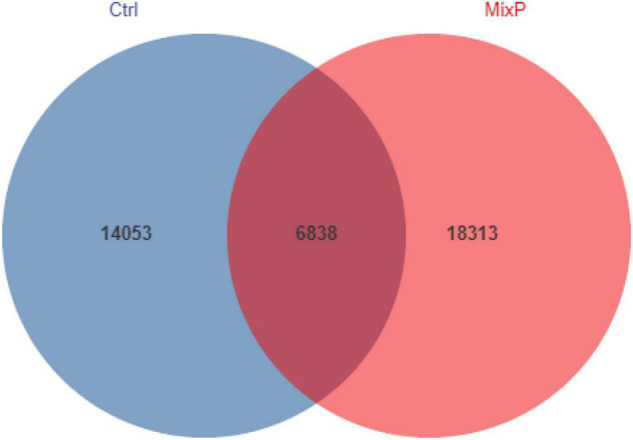
Shared operational taxonomic unit (OTU) analysis of the different groups. Each circle in the figure indicates a group, and the numbers in the circle and circle overlap represent the number of OTUs between the groups. The number not in overlap indicates the number of unique OTUs in the group.

A total of 1,150,947 bases were obtained from 16 samples. After quality inspection and removal of chimeric sequences, the average number of readings produced from each duck’s cecal sample was 40,541, with an estimated good coverage of greater than 99.6% for all cecal samples. Rare OTUs (< 0.005% of all OTUs) were removed. A total of 39,204 OTUs were obtained and successfully classified to the domain level using classifiers, as shown in Figure 1. There were 6,828 OTUs in the MixP and the Ctrl, and the number of OTU in the MixP was higher than that in Ctrl, indicating that the probiotics preparation interfered with the species and quantity of cecal flora of Shaoxing ducks.

#### Microbial Community Structure at Phylum and Genus Levels

**FIGURE 2:**
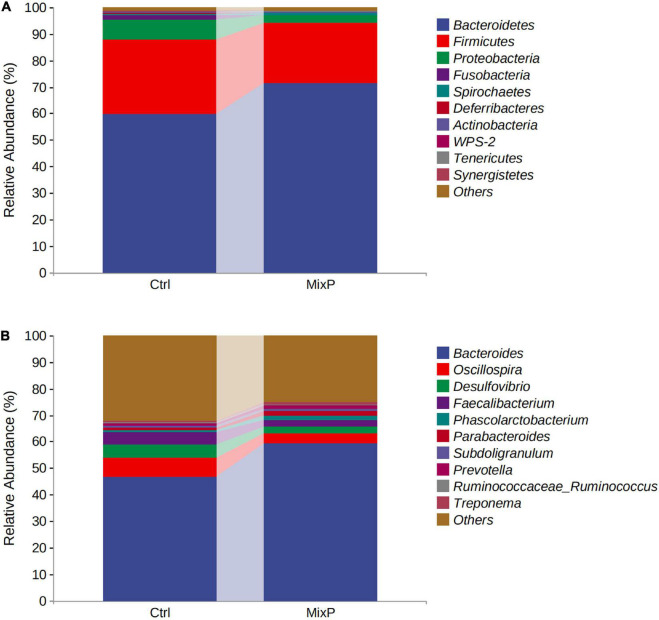
Percentage composition of the top 10 predominant phyla and genus in the cecal content. **(A)** Phylum level; **(B)** genus level.

Ten phyla of cecal microflora of duck were identified in this experiment (Figure 2A and Table 2). At the phylum level, all content samples from the test and control groups showed nearly identical community structure. In Ctrl, *Bacteroidetes* were the most abundant bacteria (71.37%), followed by *Firmicutes* (22.64%) and *Proteobacteria* (2.89%). The abundance of *Bacteroidetes* in cecal contents of Shaoxing ducks in MixP was higher than that in Ctrl (*P* < 0.05), the abundance of *Firmicutes* in cecal contents of Shaoxing ducks in MixP was lower than that in Ctrl (*P* < 0.05).

**TABLE 2:** Abundance of the major phylum in each group.

Items	Treatment	
	Ctrl	MixP
*Bacteroidetes*	59.58 ± 8.8*^b^*	71.37 ± 7.52*^a^*
*Firmicutes*	28.41 ± 1.47*^a^*	22.64 ± 1.31*^b^*
*Proteobacteria*	7.42 ± 0.13	2.89 ± 0.08
*Fusobacteria*	1.52 ± 0.22	0.27 ± 0.08
*Spirochaetes*	0.53 ± 0.19	0.76 ± 0.15
*Deferribacteres*	0.46 ± 0.11	0.18 ± 0.07
*Actinobacteria*	0.34 ± 0.19	0.29 ± 0.16

*Ctrl, control diet; MixP, compound probiotics diet.*

*^a,b^Values within a row with different superscripts differ significantly at P < 0.05. Only the first 7 flora are listed in the table.*

At the level of genus (Figure 2B and Table 3), *Bacteroides, Oscillospira*, *Desulfovibrio*, and *Faecalibacterium* are the main categories, among which *Bacteroides* occupies the largest proportion and is decreasing successively. The abundance of *Bacteroides* and *Prevotella* in cecal contents of Shaoxing ducks in MixP was higher than that in Ctrl (*P* < 0.05), and the abundance of *Oscillospira* and *Desulfovibrio* in cecal contents of Shaoxing ducks in MixP was lower than in Ctrl (*P* < 0.05).

**TABLE 3:** Abundance of the genus in each group.

Items	Treatment	
	Ctrl	MixP
*Bacteroides*	46.82 ± 6.42*^b^*	59.44 ± 9.77*^a^*
*Oscillospira*	7.09 ± 2.79*^a^*	3.54 ± 1.86*^b^*
*Desulfovibrio*	5.18 ± 1.17*^a^*	2.48 ± 0.94*^b^*
*Faecalibacterium*	4.42 ± 0.79	2.66 ± 0.72
*Phascolarctobacterium*	0.71 ± 0.3	1.83 ± 0.19
*Parabacteroides*	1.04 ± 0.73	1.38 ± 0.61
*Subdoligranulum*	0.98 ± 0.79	1.01 ± 0.53
*Prevotella*	0.5 ± 0.15*^b^*	1.06 ± 0.53*^a^*
*Ruminococcaceae_Ruminococcus*	0.59 ± 0.28	0.61 ± 0.42
*Treponema*	0.44 ± 0.51	0.7 ± 0.75
*Others*	32.22 ± 3.63	25.29 ± 3.05

*Ctrl, control diet; MixP, compound probiotics diet.*

*^a,b^Values within a row with different superscripts differ significantly at P < 0.05.*

#### Analysis of Family Differences

**FIGURE 3:**
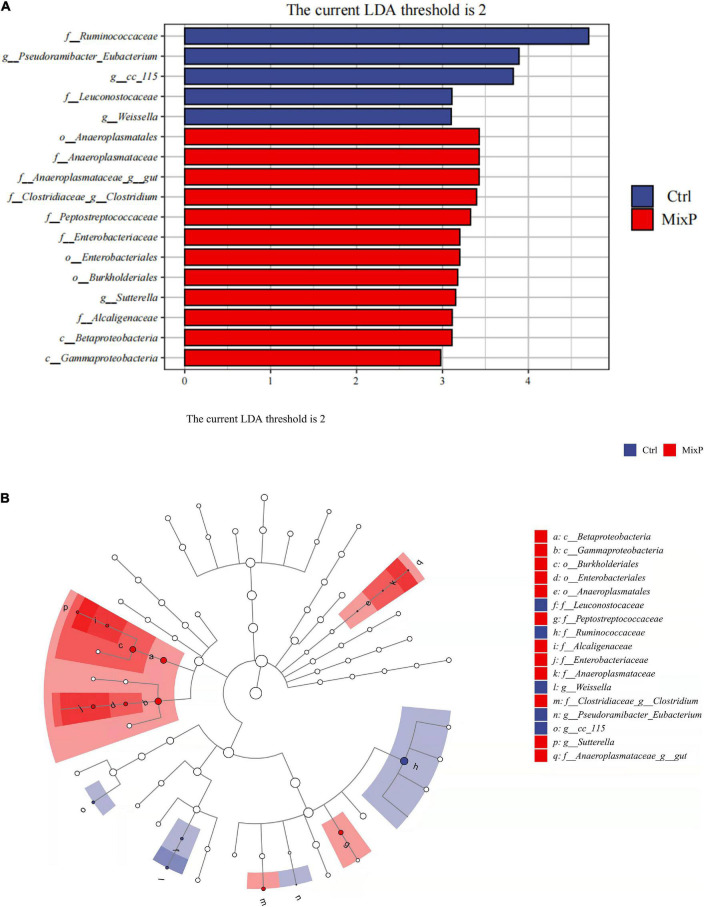
Comparison of microbial variations at the each level, using the LEfSe online tool. **(A)** Cladogram for taxonomic representation of significant differences among groups. **(B)** Histogram of the LDA scores for differentially abundant features between groups. The threshold on the logarithmic LDA score for discriminative features was set to 2.0.

LEfSe analysis results included two parts, namely, the histogram of LDA value distribution of significantly different family, which was used to show the significantly enriched family and their importance in each group (Figure 3A). Cladogram shows the taxonomic hierarchy of marker species in each group of samples (Figure 3B). The abundance of *Anaeroplasmatales* in cecal contents of Shaoxing ducks in MixP was higher than that in Ctrl (*P* < 0.05), the abundance of *Ruminococcace*in cecal contents of Shaoxing ducks in MixP was lower than that in Ctrl (*P* < 0.05).

### Metabolomic Analyses of Cecal Samples

**FIGURE 4:**
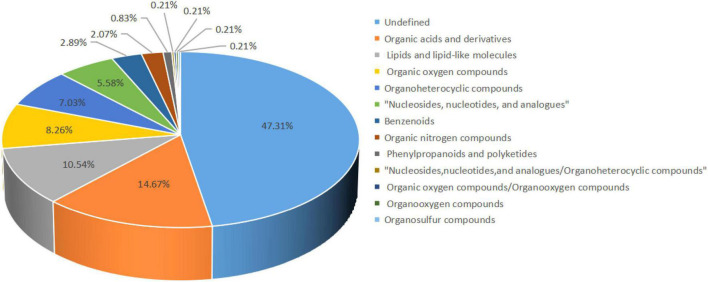
Percentage of metabolites identified. The color blocks of different colors in the figure express different chemical classification attribution entries, and the percentage represents the percentage in the chemical classification attribution entries. Metabolites without chemical classification are defined as undefined.

Our untargeted LC-MS approach assessed 484 metabolites in all samples, of which organic acids and derivatives accounted for 14.67%. Lipids and lipoid-like molecules accounted for 10.54%, organic oxygen compounds, organoheterocyclic compounds, and nucleosides, nucleotides, and analogs account for 8.26, 7.03, and 5.58%, respectively. Among them, 47.31% were undefined metabolites (Figure 4).

**FIGURE 5:**
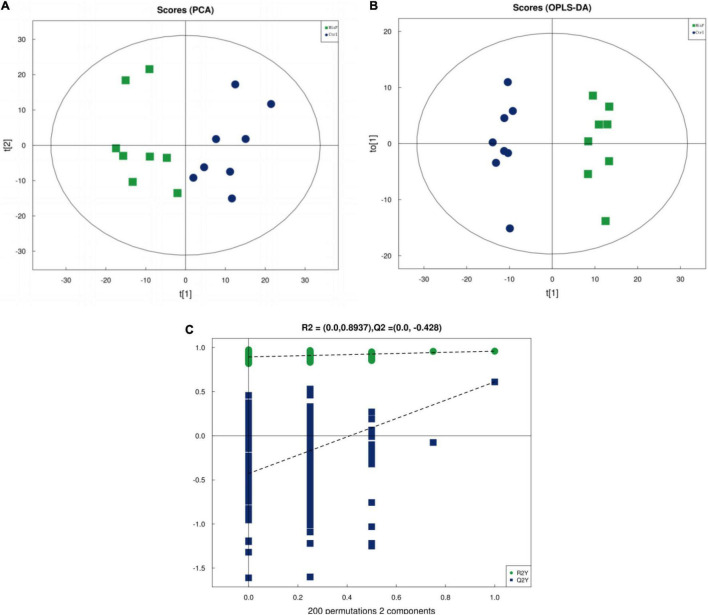
Identification of discriminating biomarkers by metabolomics analysis of ducks that received the compound probiotics diet (MixP) in comparison with the control diet (Ctrl). **(A)** The principal component analysis (PCA) and **(B)** orthogonal projections to latent structures-discriminant analysis score plots. **(C)** Permutation tests conducted with 200 random permutations in the orthogonal projections to latent structures discriminant analysis model. R^2^Y (cum) and Q^2^ (cum) represent the interpret ability and predictability of models, respectively.

The PCA plot was used to visualize the trends and outliers, showed that the Ctrl group were clustered together and that the MixP group were clustered together (Figure 5A). As shown in Supplementary Table 1, R^2^X for PCA was greater than 0.5, indicating the reliability of the PCA model. To further examine the metabolic changes, OPLS-DA analysis was performed. The results showed that the R^2^ Y (cum) and Q^2^ (cum) of the OPLS-DA were greater than 0.5 (Supplementary Table 1), indicating that the model yielded stable and accurate predictions. From the OPLS-DA score plots, the contents of cecal samples were completely separated (Figure 5B). In addition, the Q^2^ on the Y-axis was negative (−0.39) in the random-permutation test (Figure 5C), indicating that the models had good predictability and did not overfit.

**FIGURE 6:**
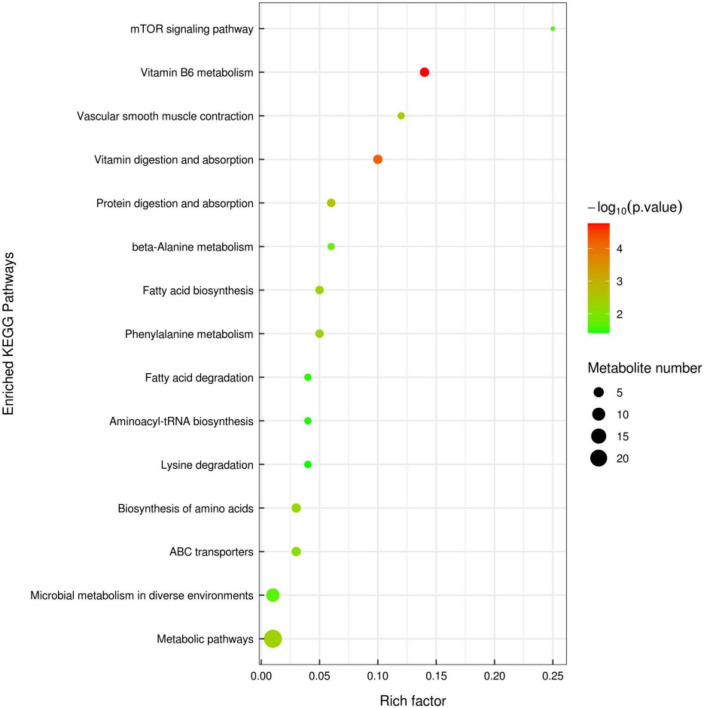
Cecal metabolomics pathway analysis of ducks that received the compound probiotics diet (MixP) in comparison with the control diet (Ctrl). The colors and sizes of the shapes represent the effects of the compound probiotics treatments on sample metabolism relative to the control treatments; larger red shapes indicate a greater effect on the pathway.

A total of 71 metabolites (38 in the negative mode and 33 in the positive mode) in the contents of cecal changed significantly between the MixP and Ctrl groups (Supplementary Table 2). Fifteen metabolic pathways were generated from the significantly altered metabolites between the MixP and Ctrl groups such as Vitamin B6 metabolism, Vascular smooth muscle contraction, Vitamin digestion and absorption, and Protein digestion and absorption (Figure 6).

### Correlation Between the Cecal Microbiome and Metabolome

**FIGURE 7:**
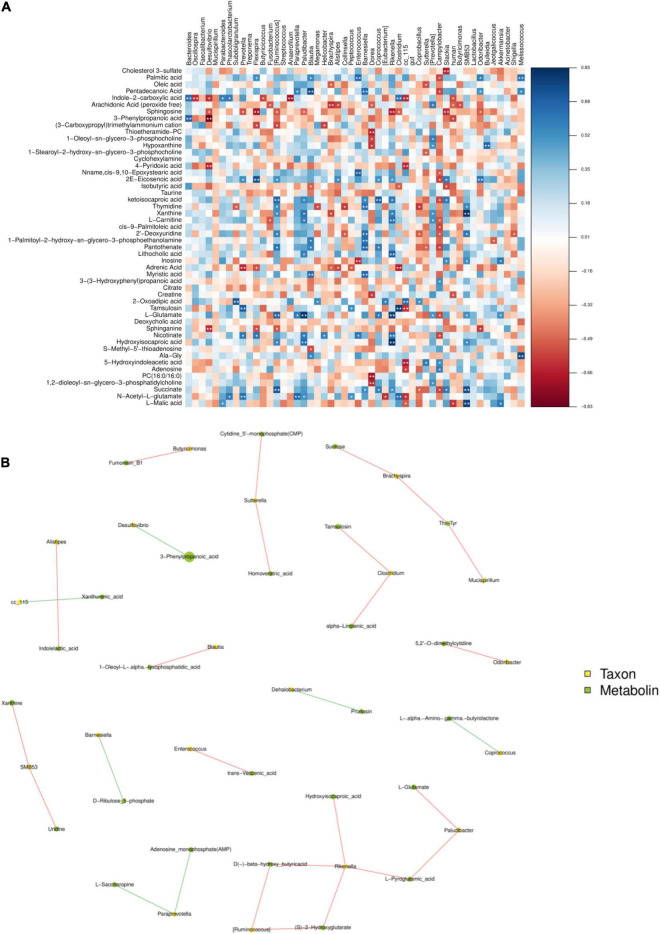
The figure is the heat map of correlation between microbial species and metabolome detection results. If the correlation is positive, it will be shown in blue; otherwise, it will be shown in red. Color depth indicates the strength of correlation; * indicates “species-metabolites” with significant association (*P* < 0.05) **(A)**. The connection between nodes indicates the existence of correlation, red line indicates positive correlation, blue line indicates negative correlation; the more connections through a node, the more information about the flora associated with it **(B)**.

The functional correlations between the different microbial genera and the altered cecal metabolites were obtained through a correlation analysis based on Spearman’s correlation coefficient values (Figure 7A). The analysis revealed high correlations (*P* < 0.05, *r* > 0.70) between several specific cecal bacteria and typical metabolites. The relative abundances of the genera *Rikenella* and Hydroxyiso Gaproic-acid D-beta-hydroxy-butyric-acid, L-Pyrogtutamic-acid and 2-hydroxyglutarate were positively correlated with L-glutarate (*P* < 0.05), *Rikenella* and *Ruminocoeeus* were positively correlated with each other (*P* < 0.05). *Desulfovibrio* has a very significant negative correlation with 3-Phenylpropanoic-acid (0.05) (Figure 7B). These data indicated that cecal bacterial compositions specifically affected cecal metabolites.

## Discussion

Cecum is the main site of fermentation in the gastrointestinal tract. It has a diverse microbial community (Zhao et al., 2013). The cecum of poultry is the main site for microbial colonization and plays a key role in the body health and intestinal development of poultry (Liu et al., 2016). Studies have shown that early intervention and colonization of intestinal flora can have a significant impact on growth performance and health of broilers (Sears, 2005; Peng et al., 2016). In Yin et al. (2010) and Gong et al. (2020, 2019), early intervention was carried out through the administration of cecal content fermentation broth, in which the complex probiotics were first colonized in the intestinal tract to precisely exert the probiotic effect, which is regarded as a new strategy for regulating the growth and body health of poultry. In the present experiment, feeding probiotics preparation from 1 day old was beneficial to the early colonization of probiotics, and the OTU number of intestinal flora in MixP was significantly higher than that in Ctrl.

The diversity of relevant microbial communities in the host gut is not a simple overview of “more diversity is better.” In short, a moderate degree of diversity is the ideal state for a stable gut microbiome. Not all microbes in the gut are beneficial, some are pathogenic, and some are conditioned. There are also some bacteria that have little effect on the host, which together constitute the diversity and richness of intestinal flora (Greyson-Gaito et al., 2020). The relative abundance values of major phyla vary greatly among different reports (Angelakis and Raoult, 2010; Wang et al., 2018). In particular, with the decrease of bacterial classification level, the similarity of reports on the relative abundance of bacterial genera and species is lower among different studies (Zhao et al., 2018). The main intestinal flora of poultry include *Firmicutes, Bacteroides*, and *Proteobacteria* (Litvak et al., 2017; Yadav and Jha, 2019), which is consistent with the results of this experiment. In this experiment, the dominant bacterial communities of the probiotic group and the control group were *Bacteroidetes*, *Firmicutes*, and *Proteobacteria* for both. The abundance of *Bacteroidetes* in cecal contents of Shaoxing ducks in MixP was higher than that in Ctrl. *Bacteroides* caecigallinarum can produce acetic acid and succinic acid as the main end products, which is one of the normal flora of intestinal contents (Saputra et al., 2015). At the genus level, *Bacteroides*, *Oscillospira*, *Desulfovibrio*, and *Faecalibacterium* are the main categories, among which *Bacteroides* occupies the largest proportion and is decreasing successively. The addition of probiotics in poultry cultivation can effectively change the intestinal microbial structure of poultry, improve the species of probiotics in intestinal flora, and reduce the species of pathogenic bacteria (Baldwin et al., 2018), which is consistent with the results of this study. In this experiment, the abundances of *Oscillospira* and *Desulfovibrio* flora in the cecal contents of Shaoxing ducks in the MixP were significantly lower than those in Ctrl. *Desulfovibrio* is known as a sulfate-reducing bacterium. In addition, it was reported that has increased significantly in DSS and FHD induced animal model (Jing et al., 2018; Li et al., 2019). LEfSe’s analysis results further refined this result from the perspective of families. Overall, the results demonstrate the usefulness of compound probiotics ecological community interaction in the protection of intestinal microbiota of Shaoxing ducks.

Metabolomics technology can be used for simultaneous detection and qualitative analysis of metabolites of small and medium molecules (MW < 1,000) in the body, organ, or tissue, and can directly and accurately reflect the changes of metabolic responses of organisms under the effects of internal and external environments (Lao et al., 2009). However, a large majority of metabolites discovered during untargeted metabolic profiling remain unknown, including many microbial compounds (Garg et al., 2017), environmental compounds (Bloszies and Fiehn, 2018), and natural compounds. The effects of compound probiotics on the metabolome of cecal contents of Shaoxing ducks were studied by LC-MS/MS mass spectrometry. The compound probiotics used are composed of *Bacillus subtilis* and *Bacillus licheniformis*. In Ducks, *Bacillus subtilis* is the strain most commonly used as a probiotic (Rajput et al., 2012; Vasaï et al., 2014). PCA results showed that there was no overlap between the two groups, indicating that the metabolic types were not similar. The results of OPLS-DA analysis also showed that probiotics had a significant effect on the metabolism of Shaoxing ducks.

Vitamin B6 (VB6) used was a pyridine derivative. It plays an important auxiliary role in neurotransmitters, amino acids, nucleic acids, glucose metabolism, and antioxidant stress, and may also regulate the expression of certain hormone receptors (Johnstone et al., 2019; Ramos et al., 2019), as a co-factor for more than 150 enzymes, has been reported to be involved in amino acid, glucose, and lipid metabolism (Gholizadeh et al., 2020). In our study, compound probiotics supplement increased the levels of vitamin B6 metabolism, and the up-regulated levels of vitamin B6 and its metabolites may indicate increased nutrient digestibility. Proteins are digested in the small intestine and broken down into free amino acids, which are then absorbed by epithelial cells into host cells. Studies have shown that amino acid utilization by epithelial cells or intestinal bacteria plays a key role in regulating intestinal homeostasis. Thus, an adequate supply of amino acids is a key factor for healthy intestinal function (Beaumont and Blachier, 2020). KEGG analysis was performed on the metabolic differential products obtained from the analysis, and the differential metabolites were mainly enriched in 15 metabolic pathways. This study mainly focused on the metabolic pathways of Vitamin B6 metabolism, Vascular smooth muscle contraction, Vitamin digestion, and absorption and Protein digestion, indicating that the supplementation of compound probiotics in Shaoxing ducks’ diet mainly affected the vitamin-related metabolic pathways.

## Conclusion

The results show that adding compound probiotics in the diet of Shaoxing ducks can significantly increase the number of OTUs and the abundance of *Bacteroidetes*, and reduce the abundance of *Oscillospira* and *Desulfovibrio* 28 different metabolites were detected. Fifteen metabolic pathways had positive effects on the intestinal tract of Shaoxing duck. The results of this experiment will provide basic data for scientific research and regulation and management of intestinal microflora in Shaoxing duck production.

## Data Availability Statement

The datasets presented in this study can be found in online repositories. The names of the repository/repositories and accession number(s) can be found below: https://ngdc.cncb.ac.cn/search/?dbId=&q=PRJCA007501.

## Ethics Statement

The animal study was reviewed and approved by Generated Statement: The experiment was approved by the Animal Care Committee of Anhui Agricultural University (no. SYDW-P20190600601).

## Author Contributions

HS prepared the manuscript and collected some data. TZ, GL, ZT, XD, and SR collected the samples. LL and WX were responsible for the design and direction of the experiment. All authors read and approved the final version of the manuscript.

## Conflict of Interest

SR was employed by the company Shaoxing Xianheng Shao Duck Breeding Co., Ltd. The remaining authors declare that the research was conducted in the absence of any commercial or financial relationships that could be construed as a potential conflict of interest.

## Publisher’s Note

All claims expressed in this article are solely those of the authors and do not necessarily represent those of their affiliated organizations, or those of the publisher, the editors and the reviewers. Any product that may be evaluated in this article, or claim that may be made by its manufacturer, is not guaranteed or endorsed by the publisher.

## Footnotes

1
https://readthedocs.org/projects/cutadapt/

2
http://www.drive5.com/usearch/manual/uchime_algo.html

3
http://drive5.com/uparse/

4
https://www.arb-silva.de/

5
https://greengenes.secondgenome.com/
