# Supplementary material for: Effects of Compound Probiotics on Cecal Microbiome and Metabolome of Shaoxing Duck
Source: Front Microbiol. 2022 Jan 11;12:813598. doi: 10.3389/fmicb.2021.813598 (PMC8787150; doi:10.3389/fmicb.2021.813598)
Supplement: Supplementary file 1 [file Data_Sheet_1.docx]

***Effects of compound probiotics on intestinal microflora and metabolome of Shaoxing duck***

Hanxue SUN Xizhong DU Tao ZENG Shenggang RUAN Guoqin LI Zhengrong TAO Wenwu XU Lizhi LU

**Frontiers in Microbiology**

**Supplementary Materials**

**Table S1**

Parameter values of principal component analysis (PCA) and orthogonal projections to latent structures-discriminant analysis (OPLS-DA) models used for the exprimental ducks.

| Type | R^2^X(cum)^1^ | R^2^Y(cum)^2^ | Q^2^(cum)^3^ |
| --- | --- | --- | --- |
| PCA score plot | 0.563 |  |  |
| OPLS-DA score plot | 0.27 | 0.975 | 0.757 |

^1,2^R^2^Y(cum) and R^2^Y(cum) represent the interpretability of models.

^3^Q2(cum) represents the predictability of models.

**Table S2**

Identification of significantly different cecum metabolites of lambs between the MixP and Ctrl groups.

| **adduct** | **Name** | **VIP^1^** | **FC^2^** | **p-value** | **MZ^3^** | **R.T^4^(s)** |
| --- | --- | --- | --- | --- | --- | --- |
| **Negative** |  |  |  |  |  |  |
| (M-H)- | 1-Palmitoyl-2-hydroxy-sn-glycero-3-phosphoethanolamine | 6.610942 | 2.949178 | 0.000379 | 452.27732 | 205.857 |
| (M-H)- | 4-Pyridoxic acid | 11.056787 | 0.170035 | 0.000422 | 182.04581 | 47.524 |
| (M-H)- | Phenylpyruvate | 1.438344 | 5.106544 | 0.000982 | 163.04053 | 58.58 |
| (M-H)- | Benzoic acid | 2.233605 | 2.802219 | 0.001637 | 121.02921 | 122.85 |
| (M-H)- | 2-Methylbenzoic acid | 1.998646 | 2.498218 | 0.002257 | 135.04486 | 129.6705 |
| (M-H)- | Homocitrate | 1.102917 | 0.337142 | 0.005259 | 205.03527 | 469.042 |
| (M-H)- | Myristic acid | 4.506957 | 2.0554 | 0.006674 | 227.20181 | 48.6185 |
| (M-H)- | 5-Hydroxyindoleacetic acid | 6.818422 | 0.015866 | 0.008415 | 190.05117 | 219.733 |
| (M-H)- | Oleic acid | 18.624546 | 2.030637 | 0.011402 | 281.24871 | 44.721 |
| (M-H)- | cis-9-Palmitoleic acid | 5.108588 | 2.11217 | 0.013185 | 253.21759 | 47.451 |
| (M-H)- | Glutaric acid | 1.298419 | 0.302519 | 0.014261 | 131.03507 | 355.842 |
| (M-H)- | Acetyl-DL-Leucine | 1.323308 | 3.725779 | 0.017609 | 172.0978 | 194.858 |
| (M-H)- | Ethylmalonic acid | 1.048406 | 0.278194 | 0.022655 | 131.03492 | 390.225 |
| (M-H)- | 3-Phenylpropanoic acid | 9.673073 | 0.565011 | 0.026076 | 149.06073 | 93.819 |
| (M-H)- | Nname,cis-9,10-Epoxystearic acid | 7.962071 | 2.572002 | 0.026241 | 297.24431 | 50.665 |
| (M-H)- | Propionic acid | 2.004114 | 0.668646 | 0.027362 | 73.02946 | 153.25 |
| (M-H)- | N-Acetyl-DL-methionine | 1.766512 | 2.178977 | 0.028253 | 190.05429 | 200.155 |
| (M-H)- | Tamsulosin | 5.637171 | 0.30687 | 0.028765 | 407.17441 | 94.546 |
| (M-H)- | Taurochenodeoxycholate | 1.653541 | 1.98756 | 0.033825 | 498.29134 | 157.164 |
| (M-H)- | Pantothenate | 4.181752 | 1.736034 | 0.036155 | 218.10368 | 276.257 |
| (M-H)- | 20-HETE | 1.35504 | 0.611805 | 0.039991 | 319.22824 | 67.442 |
| (M-H)- | L-Gulonic gamma-lactone | 1.534063 | 3.745727 | 0.042073 | 177.04055 | 94.53 |
| (M-H)- | Gentisic acid | 2.377338 | 0.173514 | 0.044377 | 153.01941 | 74.4475 |
| (M-H2O-H)- | 3-Hydroxykynurenine | 0.885979 | 0.52859 | 0.044016 | 205.06203 | 109.462 |
| (M-H)- | Anthranilic acid (Vitamin L1) | 0.789494 | 1.598377 | 0.04796 | 136.04025 | 95.881 |
| (M-H)- | Cholic acid | 0.503596 | 2.423597 | 0.01712 | 407.27967 | 254.493 |
| (M-H)- | Cytidine 5'-monophosphate (CMP) | 0.337109 | 0.510518 | 0.020719 | 322.04597 | 468.5485 |
| (2M-H)- | D-Fructose | 0.71187 | 0.372897 | 0.001862 | 359.11806 | 304.71 |
| M- | D-Lyxose | 0.459908 | 0.250653 | 0.001142 | 150.05564 | 271.1065 |
| (M-H)- | D-Ribose | 0.665006 | 0.62751 | 0.028627 | 149.04524 | 211.954 |
| (M-H)- | Fumonisin B1 | 0.715978 | 0.429862 | 0.028684 | 720.38458 | 445.4365 |
| (M-H)- | Homoveratric acid | 0.630902 | 0.38992 | 0.040842 | 195.06752 | 42.781 |
| M- | Indoxyl sulfate | 0.13591 | 1.430782 | 0.031248 | 213.00802 | 245.127 |
| (M-H)- | Isomaltose | 0.830577 | 0.370767 | 0.026617 | 341.10869 | 392.589 |
| (M-H)- | N-Acetyl-L-alanine | 0.356681 | 1.658931 | 0.049723 | 130.0507 | 254.8615 |
| (M-H)- | Stearic acid | 0.861667 | 1.974892 | 0.019298 | 283.265 | 101.829 |
| (M-H)- | Uric acid | 0.512246 | 3.677964 | 0.002363 | 167.02089 | 333.836 |
| (M-H)- | Uridine 5'-monophosphate (UMP) | 0.361445 | 0.502242 | 0.040966 | 323.02902 | 448.24 |
| **Positive** |  |  |  |  |  |  |
| (M+H)+ | Pyridoxal (Vitamin B6) | 2.457852 | 0.411555 | 0.000013 | 168.06408 | 92.514 |
| M+ | Citramalic acid | 2.691099 | 0.259195 | 0.000429 | 148.038 | 48.681 |
| (M+H)+ | 1-Stearoyl-2-hydroxy-sn-glycero-3-phosphocholine | 14.171379 | 3.030587 | 0.000518 | 524.36869 | 198.006 |
| (M+CH3COO+2H)+ | Betaine aldehyde | 2.461643 | 0.156946 | 0.00079 | 162.11143 | 307.9085 |
| (M+H-H2O)+ | 1-Palmitoylglycerol | 2.825754 | 2.250949 | 0.001882 | 313.27192 | 159.55 |
| (M+H)+ | 1-Myristoyl-sn-glycero-3-phosphocholine | 4.463387 | 3.244645 | 0.002033 | 468.30636 | 204.533 |
| (M+H)+ | Pyridoxine | 2.929986 | 0.335545 | 0.002117 | 170.07974 | 107.6 |
| (M+H)+ | Indole-2-carboxylic acid | 28.230018 | 0.182608 | 0.004151 | 162.05442 | 66.056 |
| (M+H)+ | Oxyquinoline | 5.556423 | 0.04425 | 0.004487 | 146.05909 | 220.251 |
| (M+H)+ | L-.alpha.-Amino-.gamma.-butyrolactone | 1.5147 | 0.15936 | 0.004598 | 102.05337 | 90.419 |
| (M+H)+ | Quinaldic acid | 2.877105 | 0.042837 | 0.008103 | 174.05373 | 219.13 |
| (M+H-H2O)+ | Cholecalciferol (Vitamin D3) | 1.171584 | 1.395267 | 0.011968 | 367.33342 | 37.101 |
| M+ | L-Arginine | 2.218414 | 0.285846 | 0.014271 | 174.11091 | 257.702 |
| (M+NH4)+ | Cellobiose | 1.191037 | 0.328582 | 0.018095 | 360.14785 | 392.94 |
| (M+H)+ | L-Pipecolic acid | 2.285942 | 0.290342 | 0.018364 | 130.08497 | 277.819 |
| (M+H)+ | Xanthurenic acid | 2.091306 | 0.296547 | 0.019128 | 206.04309 | 273.22 |
| (M+H)+ | Pyridoxamine (PM) | 1.434723 | 0.550476 | 0.021114 | 169.09526 | 359.642 |
| (M+H)+ | Adenosine | 4.676729 | 0.425474 | 0.029058 | 268.10334 | 172.019 |
| (M+H)+ | L-Palmitoylcarnitine | 3.921 | 2.135633 | 0.038179 | 400.34037 | 180.616 |
| (M+H)+ | Stearoylcarnitine | 5.431853 | 2.959551 | 0.038208 | 428.37161 | 177.143 |
| (M+H)+ | L-Histidine | 3.155985 | 2.015412 | 0.044895 | 156.07536 | 463.092 |
| (M+H-H2O)+ | 1-Stearoyl-rac-glycerol | 0.544833 | 3.748177 | 0.002207 | 341.30234 | 250.678 |
| (M-2H+3K)+ | (2E,6E)-Farnesol | 0.558235 | 1.606834 | 0.015468 | 337.23402 | 103.397 |
| (M+H-H2O)+ | Cer(d18:1/18:1(9Z)) | 0.239842 | 2.962351 | 0.040925 | 546.51959 | 141.486 |
| (M+CH3CN+H)+ | Dacarbazine | 0.913467 | 0.445364 | 0.010331 | 224.12578 | 245.412 |
| M+ | Glycerophosphocholine | 0.954877 | 0.359549 | 0.022833 | 258.10764 | 425.151 |
| (M+H)+ | His-Phe | 0.367942 | 0.687853 | 0.016367 | 303.14114 | 387.682 |
| (M+H)+ | Ile-Gly | 0.489982 | 0.470566 | 0.015451 | 189.12534 | 208.478 |
| (M+NH4)+ | Maltopentaose | 0.33848 | 0.290699 | 0.008157 | 846.3041 | 511.787 |
| (M+H)+ | N1-Acetylspermidine | 0.311259 | 0.812163 | 0.046983 | 188.17395 | 309.2435 |
| (M+H)+ | N-Carboxyethyl-.gamma.-aminobutyric acid | 0.587821 | 0.550161 | 0.026551 | 176.09034 | 379.6095 |
| (M+H)+ | Nicotinamide adenine dinucleotide (NAD) | 0.399966 | 0.495437 | 0.033063 | 664.11262 | 453.042 |
| (M+H)+ | Tyr-Gln | 0.452424 | 0.562291 | 0.014284 | 310.14454 | 209.865 |

Abbreviations: Ctrl = control diet; Mixp = compound probioticst.

^1^VIP >1 and ^4^*P*-value<0.05 are listed in the table. *P* -values were calculated according to Student’s T-test (n=6).

^2^FC = fold change. If the fold change value is less than 1, it means that there is less metabolite in the Mixp group than in the Ctrl group.

^3^MZ = mass-to-charge ratio.

^4^R.T = represents retention time

.
